# Contrasted TCRβ Diversity of CD8^+^ and CD8^−^ T Cells in Rainbow Trout

**DOI:** 10.1371/journal.pone.0060175

**Published:** 2013-04-02

**Authors:** Rosario Castro, Fumio Takizawa, Wahiba Chaara, Aurélie Lunazzi, Thi Huong Dang, Bernd Koellner, Edwige Quillet, Adrien Six, Uwe Fischer, Pierre Boudinot

**Affiliations:** 1 Institut National de la Recherche Agronomique, Virologie et Immunologie Moléculaires, Jouy-en-Josas, France; 2 Friedrich-Loeffler-Institut, Federal Research Institute for Animal Health, Institute for Infectiology, Greifswald-Insel Riems, Germany; 3 UPMC Univ Paris 06, UMR 7211, Paris, France; 4 Centre National de la Recherche Scientifique, UMR 7211, Paris, France; 5 Assistance Publique - Hôpitaux de Paris, Hopital Pitié Salpêtrière, Service de Biothérapie, Paris, France; 6 Institut National de la Recherche Agronomique, UMR1313 Génétique Animale et Biologie Intégrative, Jouy-en-Josas, France; University of Nebraska Medical Center, United States of America

## Abstract

Teleost fish express highly diverse naive TCRβ (TRB) repertoires and mount strong public and private clonal responses upon infection with pathogens. Fish T cells express typical markers such as CD8, CD4-1 and CD4-2, CD3, CD28 and CTLA4. Fish CD8^+^ T cells have been shown to be responsible for antigen-specific cell-mediated cytotoxicity in *in vitro* systems using histo-compatible effector and target cells. We compare here the complexity of TRB repertoires between FACS sorted CD8^+^ and CD8^−^ T cells from spleen and pronephros of rainbow trout. In contrast to human, while the TRB repertoire is highly diverse and polyclonal in CD8^+^ T cells of naïve fish, it appeared very different in CD8^−^ lymphocytes with irregular CDR3 length distributions suggesting a dominance of activated clones already in naïve fish or the presence of non conventional T cells. After infection with a systemic virus, CD8^+^ T cells mount a typical response with significant skewing of CDR3 length profiles. The infection also induces significant modifications of the TRB repertoire expressed by the CD8^−^ fraction, but for a different set of V/J combinations. In this fraction, the antiviral response results in an increase of the peak diversity of spectratypes. This unusual observation reflects the presence of a number of T cell expansions that rise the relative importance of minor peaks of the highly skewed distributions observed in unchallenged animals. These results suggest that the diversity of TRB expressed by CD8^+^ and CD8^−^ αβ T cells may be subjected to different regulatory patterns in fish and in mammals.

## Introduction

The adaptive immune response to infectious agents is characterized by initial priming and expansion of T and B cell clones specific of the pathogen. Antigens derived from the pathogen can be specifically recognized by the T cell antigen-specific receptor (TR), a disulfide-linked membrane-bound heterodimer expressed by T lymphocytes. TR comprises two chains, either α/β or γ/δ, each composed of two immunoglobulin superfamily domains (V for variable and C for constant), and a very short intra-cytoplasmic tail. The variable domain is highly diverse due to somatic rearrangements in variable (V), joining (J), and, in the case of the β or δ-chain, diversity (D) segments, occurring during T cell differentiation. This large diversity allows a specific recognition of any antigen by a few T cell clones in an individual, leading to activation and clonal expansion. The dynamics of antigen-specific lymphocyte responses *in vivo* during the course of infection follows a general pattern: the initial expansion of effector cells precedes a rapid contraction phase, leaving a relatively stable, small pool of memory cells that provide long-term immunity.

In mammals, protein antigens are recognized by αβ T cells as short peptides exposed at the surface of antigen (Ag)-presenting cells by Major Histocompatibility complex (MHC) molecules [1]. CD4 and CD8 co-receptors bind the side of MHC molecules on antigen presenting cells, and thereby increase their signalling capacity by intracellular recruitment of the lymphocyte specific kinase (LCK) [2]. These co-receptors determine on which class of MHC molecules αβ TRs recognize their specific peptides: CD4^+^ T cells target peptides presented on MHC class II while CD8^+^ T cells target peptides presented by the MHC class I. Once primed, CD4^+^ T cells migrate to follicles to help B cells produce antibodies, and to peripheral sites of antigen exposure to fight incoming pathogens by inducing the appropriate type of effector cell function. CD4^+^ T cells regulate the immune response through cytokine secretion and can be subdivided into different categories including regulatory CD25^+^CD4^+^ T cells, and helper T cells with various profiles of cytokine production [3], [4]. Type 1 effector CD4^+^ T helper (Th)-1 cells produce IFN-γ that promotes clearance of viruses and intracellular bacteria, while type 2 CD4^+^ Th2 cells produce IL-4, IL-5 and IL-13 that promote clearance of extracellular parasites. Another subset named Th17 is characterized by the capacity to produce IL-17, IL-21 and IL-22 and plays a key role in inflammation. Once antigen is eliminated, central memory and effector memory T cells persist in the memory pool to provide systemic immune surveillance in secondary lymphoid and in non-lymphoid tissues, to react promptly in case of secondary infection. CD8^+^ T lymphocytes possess cytotoxic capacity and are responsible for the elimination of virus-infected and tumor cells. Following their initial expansion and subsequent clearance of the viral infection, most cytotoxic T lymphocytes (CTL) undergo apoptosis, leaving behind a small but stable pool of memory CD8^+^ T cells. Thus, after early double-positive thymocytes express both CD4 and CD8 molecules and undergo differentiation into either CD8^+^CD4^−^ or CD8^−^CD4^+^ single-positive cells, CD4 and CD8 distinguish two basic lineages of αβ T lymphocytes representing T cells with heterologous functions [5].

Basic subsets of αβ T cells characterized by the expression of CD4 and CD8 have been found also in fish, indicating that these markers constitute fundamental molecules of the vertebrate T cell response, together with classical MHC class I and II, CD3, CD28 and CTLA4 co-receptors. In teleost fish, CD8α was discovered first in rainbow trout where it was expressed at high levels in the thymus and at lower levels in spleen, kidney, gut, and blood leukocytes [6]. CD8α and β were later found in a number of other teleost fish species, and also in Chondrichtyans [7]–[10]. Although trout CD8α lacks a typical LCK binding motif in its cytoplasmic tail, it has been recently shown that the CD8 co-receptor can bind LCK as in mammals [11]. Importantly, the expression level of CD8 transcripts increases during infections and allogenic stimulation [12], and is correlated with lymphocyte cytotoxic activity in ginbuna crucian carp [13]. Alloantigen-specific cytotoxicity is mediated by CD8^+^ cells, but not by CD8^−^ cells in the same species [14]. Also, several CD8^+^ channel catfish leukocyte cell lines were identified as allospecific effectors, providing further evidence for the existence of cytotoxic T cells in teleosts [15]. Collectively, these observations indicate that cytotoxicity reported a long time ago in fish in graft experiments is mediated by CD8^+^ T cells [16]. Regarding CD4, two copies have been found in fish: *cd4-1*, encoding a receptor with 4 Ig domains as in mammals, and *cd4-2*, encoding 2 Ig domains [17], [18]. Both CD4-1 and -2 are mainly expressed by CD8^−^IgM^−^ lymphocytes, and their intracellular regions possess the canonical CXC motif required for the LCK binding, suggesting they are involved in similar mechanisms of T cell signaling as in mammals. Orthologues of the cytokines promoting the differentiation and effector functions of Th1 and Th17 cells have been found in fish including IL-2, IL-10, IL-12, IL-18, IFNα, IFNγ and IL-17, respectively, as well as the corresponding key transcription factors EOMES and T-bet [19], [20]. Additionally, Th2 transcription factors GATA3 and STAT6 have been identified in several fish species [21]–[24], as well as Th2 cytokines belonging to the IL4/IL13 family [24]. These findings suggest that fish express a complex array of cytokines that has the capacity to regulate different functional types of CD4^+^ T cells reminding mammalian Th1 and Th17. Hence, several lines of evidence indicate that CD8 and also probably CD4-1 and -2 identify two main lineages of T cells with conserved functions in vertebrates, at least since the common ancestor of teleost fish and mammals.

However, very little is known about the respective TR repertoires of CD8^+^ and CD8^−^ (including CD4^+^) T cells in fish, and their respective responses to pathogens. We previously found that the TRβ (TRB) repertoire expressed by rainbow trout spleen leukocytes was diverse and polyclonal in healthy animals, and that viral infection induced a complex arrays of private responses and a public specific response retrieved in all clonal individual fish studied [25]. The TR diversity expressed by the different T cell subsets, which remains unknown, would be important to identify what arm(s) of T cell immunity is triggered during immune responses. Thus, a system set-up to follow the modifications of the TR repertoire expressed by CD8^+^ versus CD8^−^ T cells, would open the way to the characterization of the clonal complexity of cytotoxic and helper or regulatory responses, respectively. It would therefore lead to an accurate modeling of the impact of the route of infection, the adjuvant, the nature of Ag, and to a better understanding of the contribution of fish T cell immunity to the protection afforded by vaccination.

In primates, CD4^+^ and CD8^+^ αβ T cell repertoires of peripheral blood mononuclear cells (PBMC) are roughly of equal complexity, although it is generally accepted that the CD8^+^ T cell subset is responsible for the majority of the observed skewing of complementary determining region (CDR)-3 length TRB spectratypes [26], [27]. In the same line, the CD8^+^ T cell repertoire was found highly restricted during the progression of Acquired Immuno-Deficiency Syndrome (AIDS), while no perturbation of CD4^+^ T cell repertoire was obvious in the early stages of the disease [28]. Finally, both CD4^+^ and CD8^+^ T cell subsets are skewed in aging with frequent clonal proliferation observed in healthy elderly humans for a variety of TRB V (TRBV) families [29].

In this work, we compared the diversity of TRB expressed by CD8^+^ and CD8^−^ T cell subsets in rainbow trout using CDR3 length spectratyping. We show that the CD8^+^ T cell population expresses a diverse polyclonal repertoire, which is skewed after response to a systemic viral infection as previously described from unsorted spleen leukocytes in [25], [30]. In contrast, CD8^−^ cells express a lower diversity of TRB with high inter-individual variations, even between naïve clonal individuals. Our observations suggest that rainbow trout spleen and pronephros contain a large repertoire of CD8^+^ T cells in naïve fish, while the CD8^−^ compartments comprise a significant proportion of expanded clones possibly produced during previous immune responses.

## Materials and Methods

### Ethics Statement

All animals were handled in strict accordance with good animal practice as defined by the European Union guidelines for the handling of laboratory animals (http://ec.europa.eu/environment/chemicals/lab_animals/home_en.htm) and by the Regional Paris South Ethics committee, and animal work was approved by the Direction of the Veterinary Services of Versailles (authorization number 78-28). The infection trials performed at the Friedrich-Loeffler-Institute were approved by the ethics commission of the German state Mecklenburg-Western Pomerania under the reference number LALLF M-V/TSD/7221.3-1.1-036/09.

### Fish

Heterozygous isogenic rainbow trout, clonal line A36-A3, were produced at the INRA experimental fish farm (PEIMA, Sizun, France) by crossing two double haploid fish lines and sent as hatched eggs to FLI for hatching and further rearing. This clonal line is a cross between two fully homozygous individuals belonging to two distinct clonal lines that were originally established through two successive generations of gynogenetic reproduction (suppression of mitosis and meiosis in the first and second generations, respectively) [31]. Fish were at the age of two years during sampling. They were maintained in 400-liter tanks in a partially re-circulating water system and fed with commercial dry pellets.

### Viral Challenge: Immunization Protocols and Sampling

Immunization and virus challenge were performed using the attenuated 25-111 variant of strain 07-71 of VHSV [32] through intraperitoneal injection. A first sub-lethal dose of 10^5^ plaque forming units (PFU)/fish in 100 µl of sterile, endotoxin-free PBS was applied to each of the four fish in the infected group. This infection usually leads to good protection against a subsequent lethal infection. Four weeks later, fish received a second injection of the same virus (5×10^7^ PFU/fish) and samples were collected 3 weeks later. Four control fish were injected with the same volume of PBS. Trout were sacrificed following approved procedures and subsequently exsanguinated.

### Leukocyte Preparation

All following preparation steps were performed on ice or at 4°C. Pronephros and spleen were individually removed from the peritoneal cavity and homogenized with a Potter–Elvehjem homogenizer to prepare single cell suspensions. Cells were resuspended in mixed medium (MM, Iscove’s DMEM/Ham's F12 (Gibco) at a ratio of 1∶1, supplemented with 10% fetal bovine serum (FBS)) and layered onto an isotonic Percoll (Biochrome) gradient (? gradient (rad. After centrifugation at 650×g for 40 min cells at the interphase were collected and washed twice using MM.

### Staining and Sorting of Lymphocytes

Pronephrocytes and splenocytes from individual fish each were pooled and double labelled with two monoclonal antibodies (mabs). For this purpose, a rat anti-trout CD8α mab [33] was combined with FITC-conjugated goat anti-rat IgG (H+L) (Jackson ImmunoResearch). The same cells were double stained with mab D11 labelling lymphocytes other than B cells and thrombocytes (manuscript in preparation) in combination with an R-PE conjugated F(ab’)2 donkey anti-rat IgG (H+L) (Jackson ImmunoResearch) secondary antibody. Sorting was performed with a MoFlo high-speed cell sorter (Dako Cytomation). Only single (excluding doublets through pulse width gating) lymphocyte like cells (low FSC/low SSC) were considered in the sorting decisions. Macrophage-like cells (high FSC/low SSC), granulated cells (high FSC/high SSC) and cell debris were excluded from sorting. Three subpopulations were collected (30000 cells per population): CD8^+^/D11^+^ double positive cells, CD8^−/^D11^+^ single positive cells, and double negative cells. CD8^+^/CD11^−^ single positive cells were not detected. 3×10^4^ cells of each subpopulation were directly sorted into 600 µl lysis buffer (RNeasy Micro Kit from Qiagen) containing 6 µl mercaptoethanol to prevent RNA degradation. A part of each subpopulation was sorted into MM to subsequently analyse their purity by flow cytometry at same analysis settings as for sorting.

### RNA Preparation and cDNA Synthesis

Total RNA was purified and DNase treated using QIAgen RNA extraction kit. RNA (2 µg) was reverse transcribed into cDNA using Superscript II Reverse Transcriptase (Invitrogen Life Technologies) with 2.5 µM oligodT_25_ primer in a final volume reaction of 20 µl. For sorted cells, total RNA was extracted from each cell population using RNeasy Micro Kit (Qiagen). Full-length cDNA was generated and amplified using the SMARTer™ PCR cDNA synthesis kit (Clontech Laboratoires, Inc.), following manufacturer’s instructions. The optimized protocol of this kit preferentially enriches for full-length cDNAs and retains true gene representation of genes in the original sample. The optimal number of PCR cycles determined for ds cDNA synthesis and amplification of S1, S2 and S3 samples was 23 cycles.

### CDR3 Length Spectratyping Analysis

The spectratyping of TRB CDR3 length (Immunoscope analysis) first developed for mouse or human was previously adapted for rainbow trout [25]. A first amplification (PCR1) using a forward primer specific for a subgroup or a set of TRBV genes in combination with a reverse primer TRBC specific for the constant region was performed as follows: 1 µl cDNA was used as template for PCR1 using 0.4 mM of each dNTP, 0.4 µM of each primer (forward: TRBV_family specific_, reverse: TRBC), and 0.025 u µl^−1^ of GoTaq DNA polymerase (Promega) in 1× reaction buffer with 2 mM of MgCl_2_ (95°C for 5 min; 40 cycles of 95°C 45 s, 60°C 45 s, 70°C 45 s; 70°C 10 min) (**Table 1**). Primers specific for TRBV combined with a primer specific for TRBC amplify TRB sequences with TRBV from a given family but with different TRBJ content and diverse CDR3 lengths. In a second step, TRBV-TRBC PCR products were subjected to run-off reactions (PCR2) using 5′ 6-FAM- fluorescent C internal, reverse primers, or TRBJ reverse primers. Two µl of PCR1 product were applied as template using 0.4 mM of each dNTP, 10 pmoles of the fluorescent reverse primer, and 0.025 u µl^−1^ of GoTaq DNA polymerase (Promega) in 1× reaction buffer with 2 mM of MgCl_2_. The reaction mixtures were amplified in the following conditions: 95°C for 5 min; 5 cycles of 95°C 1 min, 60°C 1 min, 70°C 2 min; 70°C 10 min. Two µl of the run-off products were mixed with 8 µl deionized formamide (Applied Biosystems) and 0.5 µl of the internal standard (GeneScanTM 500XL ROX, size standard, Applied Biosystems). The mix was then denatured at 95°C for 5 min and placed on ice before analysis in an ABI 3730HT sequencer (Applied BioSystems) at GeT-PlaGe core facility, Toulouse, France. Spectratyping data of each TRBV-TRBC and TRBV-TRBJ combinations were extracted using GeneMapper software (Applied BioSystems) and analysed with ISEApeaks software [34] [35]. Each spectratype or profile is composed of several peaks (typically 4 to 10) separated according to their corresponding length of run-off products, spaced by 3 nucleotides as expected for in-frame transcripts. Each peak corresponds to a given CDR3 length. For each profile associated with a TRBV-TRBC or TRBV-TRBJ combination, the area under each peak was calculated and stored in a peak database. These values were then computed to quantify the differences between CDR3 length distributions.

**Table 1:** Primer sequences used in this study.

Name	Sequence (5′-3′)
*Repertoire*	
TRBV1-F	AGACAGCTTCCAGGAGAGG
TRBV2-F	AAACACCTGCAGACCTGTAC
TRBV3-F	CAACAGTCAGCCCAAAACACT
TRBV4-F	AAGCCTAATGCCTCTGTCACA
TRBV5-F	ACCAGAAATCACAAGGAGAAACAG
TRBV6-F	CCAGGCTTCAGCATGCCCAGCTACA
TRBV7-F	AAGGAGATGCGTCAGTCACTCTA
TRBV8-F	AAGGACGGGCGCGAGGCGGACATC
TRBV9-F	GGCCACGAAACACCTTAAAGATG
TRBV10-F	AGCAGTCAATAGCTGATTCTAATC
TRBJ1_2-R	TAAAACAGTGAGTTTGGTTCCATT*
TRBJ2-R	CATTGCCAAAGAAGGCTGG*
TRBJ3-R	CAGAACAGTCAGTTTGGTTCCCG*
TRBJ4-R	GAGAACTGTTAATTTGGTGCCTTG*
TRBJ6-R	CCGGGTTCCTCCACCGAAGTC*
TRBJ7-R	ACGGTGAGTTTGGTGCCGG*
TRBJ8-R	TGCCGTTGCCGAAGTACG*
TRBJ9-R	GTGAGTCTGGAACCTGGA*
TRBJ10-R	ACTTCCCTCTCCAAAGTAGGC*
TRBC2-R	GTAGAAGCGGGTGGCTACAC
TRBC1-R	GTTTCTGTCTTCACACTTCTTAGC*
*qPCR*	
EF1-α-qF	CAAGGATATCCGTCGTGGCA
EF1-α-qR	ACAGCGAAACGACCAAGAGG
β-actin-qF	GGGAGAAGATGACCCAGATCATG
β-actin-qR	GGTGGTACGGCCAGAGGC
CD3γδF	CCTGATTGGAGTAGCTGTCTAC
CD3γδR	GCTGTACTCAGATCTGTCCATGC
CD4.1-F	AGCTTGAACGTGTTGCTGT
CD4.1-R	TCGAGTTACTTCACCAAACAC
CD8F	GAGTACACCTGCGCTGTGGAAT
CD8R	TCGAGTTACTTCACCAAACAC

* The 5′ 6-FAM- fluorescent version of the primer was used for immunoscope analysis.

1. In a given context, the “perturbation score” for a given TRBV-TRBC or TRBV-TRBJ profile was calculated as follows:

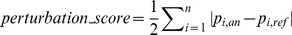
where *p_i,an_* and *p_i,ref_* are the relative areas of the peak #i from the analysed and the reference profiles, respectively; n is the number of peaks detected in the reference profile.
  An average perturbation was then computed for all the TRBV-TRBC or TRBV-TRBJ of a given sample, and/or for the different individuals of a dataset. As the intensity of CDR3 peaks was usually not comparable between different amplifications, we considered the percentage of use of each CDR3 length (i.e. the “relative area”), obtained by dividing the area of CDR3 peaks by the total area of all peaks within the profile. Profile perturbations range from 0 (analysed profile is identical to the reference), to 100 (no overlap at all).
2. The repertoire diversity can be assessed by diversity measures based on the concept of Shannon entropy that provides a measure of the quantity of information encompassed in the repertoire. Given P = (p_1_,…,p_S_): a CDR3 profile where *p_i_* is the relative proportion of the peak #i within S peaks and 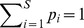.

The Shannon diversity index (SDI) is usually estimated using the following formula:
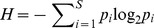



H reaches the maximum when *p_i_* (i = 1,.,S) follows a uniform distribution, whereas a maximal TRB repertoire diversity is expected for a Gaussian-like distribution of CDR3 lengths. We therefore computed a modified version of Shannon entropy which reaches its maximum for a Gaussian-like CDR3 length profiles by transforming profile P into profile Q* according to a reference profile Q = (q_1_,…,q_S_) (considered to have a maximal diversity) and applied Shannon entropy to the transformed profile Q*.

First we calculated a profile Q’(q’_1_,…,q’_S_).
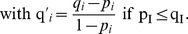


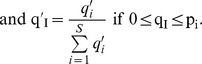



To satisfy the normalization hypothesis of Shannon entropy, we normalized the profile Q’ bydividing each relative proportion by its overall sum thus obtaining Q* with 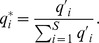


Finally, the Shannon entropy for the transformed and normalized profile Q* was computed as:
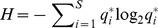



To assess a significant differences between groups, we performed statistical tests at level α = 0.01 (type 1 error) on the perturbation scores and diversity index. Statistical significances of the differences for different group pairs were determined using empirical Bayes test from the limma (Linear Models for Microarray) packages (R/Bioconductor). We chose this test because it outperforms the classical Student t-test or Mann-Whitney-Wilcoxon non parametric test for small samples [36]. In addition, repertoire perturbation scores were used to perform Principal Component Analysis (PCA) analysis in order to compare the statistical dispersion of the samples on a multidimensional plan. Statistical and multivariate analyses were performed using R software (http://www.r-project.org/).

### Real-time PCR Analysis of Gene Expression

For real time PCR, 3 µl of cDNA (1∶3 diluted) was used as a template for amplification using gene specific primers (**Table 1**). PCR amplification was performed in a Mastercycler® ep realplex (Eppendorf), using ready prepared 2× master mix (Power SYBR Green PCR master mix, Applied Biosystems) with a final PCR volume of 25 µl, in white 96-well plates (Eppendorf). PCR conditions were 95°C for 10 min followed by 95°C for 30 sec, 60°C for 30 sec and 72°C for 30 sec. The fluorescence signal output was measured and recorded at 80°C during each cycle for all wells for 40 cycles. A negative control (no template) reaction was also performed for each primer pair. QPCR calculations were based on the normalization of the expression of the genes of interest using the expression of the housekeeping gene trout elongation factor-1α (EF1-α). Equivalent EF1-α expression levels were obtained from populations S1, S2 and S3 for a given number of cells processed, confirming that the absence of expression of the T cell markers CD4, CD8 and CD3 in S3 was not due to RNA degradation. A sample from the serial dilution was run on a 2% agarose gel, stained with ethidium bromide and viewed under UV light to confirm that a band of the correct size was amplified. A melting curve for each PCR was determined by reading fluorescence every degree between 60°C and 95°C to ensure that only a single product had been amplified. EF-1α and β-actin were used for normalization of expression. The relative expression levels of the genes of interest were determined using the Pfaffl method [37]. Efficiency of the amplification was determined for each primer pair using serial 10 fold dilutions of pooled cDNA, performed in the same plate as the experimental samples. The efficiency was calculated as E = 10 ^(−1/s)^, where s is the slope generated from the serial dilutions, when Log dilution is plotted against ΔCT (threshold cycle number).

## Results

### Isolation of CD8α^+^ and CD8α^ –^ Cell Subsets

Since the expression of the rainbow trout CD8 co-receptor appears to be correlated with T cell cytotoxic activity, we undertook the analysis of the TRB-repertoire of FACS-sorted CD8^+^ and CD8^−^ cell subsets to compare the respective TR diversity of cytotoxic and other αβ T cells in fish. The primary αβ T cell repertoire is shaped by negative and positive selection exerted in the thymus during T cell differentiation. These mechanisms depend on the presentation of self-peptides by MHC molecules at the surface of thymic stromal cells. It is therefore highly restricted by the MHC alleles expressed by the individual, as well as antigen-driven T cell responses occurring in periphery. To avoid fish-to-fish genetic variability that could lead to different TRB-repertoires, we used isogenic fish – i.e. a F1 cross between two fully homozygous fish belonging to gynogenetic clonal lines, which ensures that all individuals were genetically identical at all loci [31].

**Figure 1:**
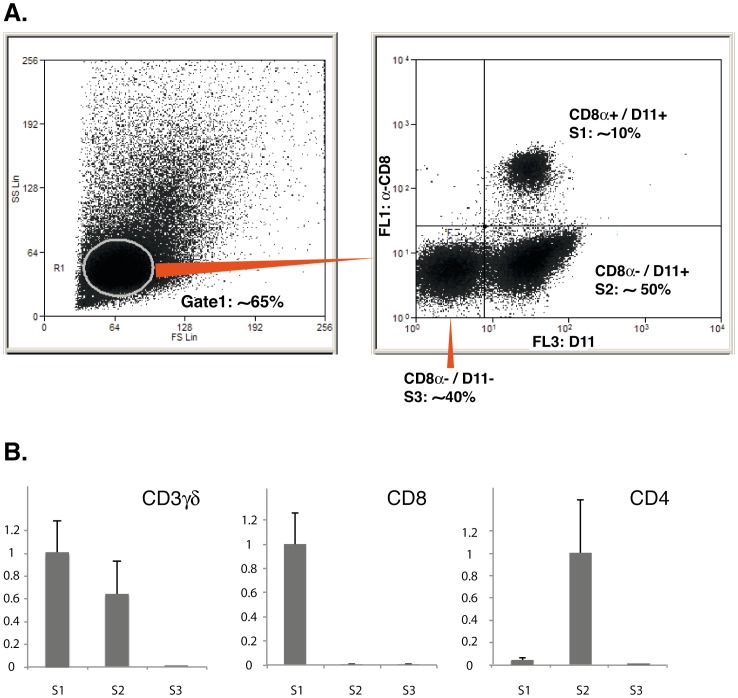
Separation of CD8α+ and other T cells. Leukocytes were prepared from spleen and pronephros by density gradient centrifugation and stained with anti-CD8 mabs. (A) A lymphocyte gate (≈65% of events) was defined, and three subpopulations were sorted from it: a CD8^+^D11^+^ population (S1), a CD8-D11^+^ population (S2), and a CD8^-^D11^-^ population (S3). Plots are from a single fish representing typical distributions of the lymphocyte subpopulations S1-3. (B) RNA and cDNA was prepared from each population (S1-3) and the expression of the T cell markers CD3γδ, CD4 and CD8α analysed by QPCR for individual fish (n = 4). The mean value is shown, and error bars represent the standard deviation among fish. The expression was normalized on the expression level of *Elf-1a* used as a housekeeping genes, and is represented as a proportion of the maximal expression level among S1-3 subsets.

For flow sorting, two mabs were used: an anti-CD8α mab previously characterized in [33] and the mab D11, a newly established mab that labels 95% of thymocytes, about 15% of PBL and about 20% of spleen leukocytes. Although the exact nature of its target molecule still remains unknown, the mab D11 labels lymphocytes others than B cells, but not thrombocytes. Moreover, D11^+^ lymphocytes from unstimulated fish do express *trb*, *cd4* and *cd8*α mRNAs but not *igm* or the thrombocyte marker *cd41* (manuscript in preparation). D11^+^ represented 60% of the gated lymphocyte-like cells, approximately 10% of which were CD8α positive. The remaining D11^−^ CD8^−^ double negative lymphocyte-like cells represented 30–40% of the gate. Since all CD8α Since all CD8^+^ there was no population of CD8α single positive cells (**Figure 1A**).

Three cell populations were sorted from the lymphocyte gate (FSC^low^/SSC): a CD8^+^D11^+^ subset (S1) containing CD8^+^ T cells only, a CD8^−^D11^+^ subset containing other lymphocytes among those most likely T helper cells (S2), and a CD8^−^D11^−^ subset containing other cells with lymphocyte scatter characteristics such as B cells and thrombocytes (S3). To validate the sorting conditions sorted subpopulations were checked by subsequent flow cytometry were purities of more than 97% were recorded (data not shown). Moreover, RT-QPCR experiments showed that the CD8α/D11 double positive lymphocytes (S1) expressed *cd3γδ* at a high level, while cells from S2 expressed about twice less, and S3 cells were *cd3γδ* ^−^ (**Figure 1B**). Accordingly, *cd8*α and *trb*, but not *cd4* transcripts were expressed in the cDNA for the S1 population while *cd4* and *trb*, but not *cd8α* transcripts were amplified from the D11 single positive cells (S2) (**Figure 1B**). The double negative cells (S3) did not express *cd8α* or *cd4* mRNA (**Figure 1B**). *Trb* transcripts were also undetectable (data not shown). Thus, the S1 population contained CD8α^+^ T cells, the S2 contained CD4^+^ and other T cells, and S3 contained all the other cells with lymphocyte morphology. Thus, the sorting procedure efficiently separated CD8α^+^ and CD4^+^ αβ T cells from trout leukocytes, allowing relevant comparisons of TRB repertoires expressed by these two cell subsets.

### CD8α^+^ and CD8α^ –^ T Cell Subsets Harbour Distinct TRB Repertoires in Naïve Fish

Previous studies have shown that “all-T cell” TRB-repertoires from lymphoid tissues of naive rainbow trout are highly diverse and polyclonal: CDR3 length spectratyping produced quasi-regular bell-shaped distributions of CDR3 length for all TRBV-TRBJ combinations from spleen, pronephros and thymus [25].

We first compared the TRB diversity of the different leukocyte fractions purified from spleen and pronephros of naïve fish using the same spectratyping approach. We studied the CDR3 length profiles of TRBV-TRBC PCR products, using an internal TVBC primer for the run-off step. This procedure integrates junctions with all TRBJ segments for a given TRBC, and provides a first synthetic measure of the diversity. These experiments generally resulted in typical bell-shaped profiles from both CD8^+^ (S1) and CD8− (S2) fractions, as displayed for TRBV1-C, TRBV4-C, TRBV8-C and TRBV10-C (**Figure S1**). Although TRBV1-C profiles appeared less regular for the CD8^−^ fraction compared to the CD8^+^ sorted cells, the difference was not significant (Wilcoxon *p* = 5.07E−02; eBayes *p* = 1.23E−01). Importantly, since the TRBV primers are specific for TRBV gene families, each TRBV-TRBC profile aggregates the distribution for different TRBV genes and all TRBJ, which can strongly buffer differences between TRBV-TRBJ combinations.

**Figure 2:**
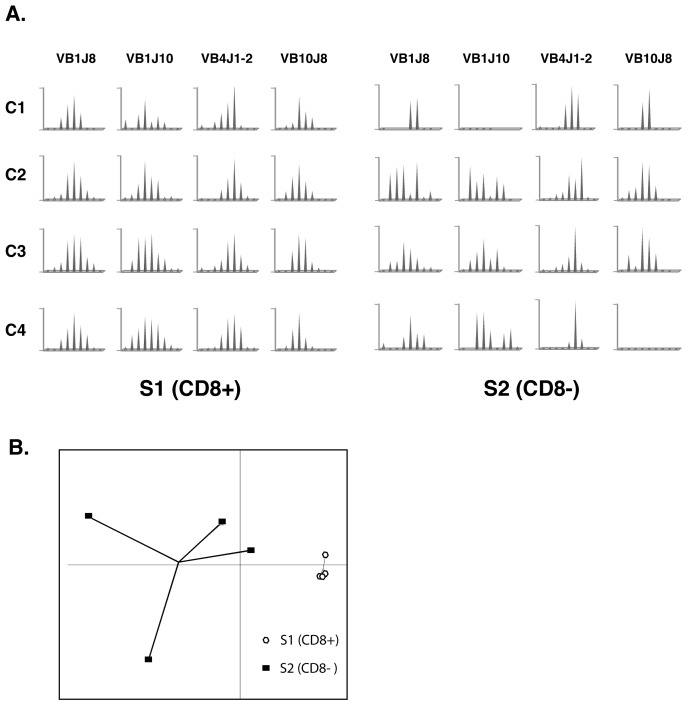
Comparison of CDR3 length spectratypes between S1 and S2 cell fractions in naïve fish. (A) CDR3 length profiles from CD8^+^ (S1) and CD8^−^ (S2) lymphocyte subsets are represented for selected TRBV-TRBJ combinations from four control fish (C1–4) (B) PCA projection of S1 and S2 samples according to the first two components (capturing respectively 58.67% and 14.94% of the global variability) using perturbation scores calculated for all spectratypes analysed.

To get a more detailed comparison, we then compared TRBV-TRBJ CDR3 length profiles between the CD8^+^ and CD8^−^ sorted fractions, using TRBJ specific primers for run-off reactions. We selected TRBV1, 4, 8 and 10 from our previous observations to study highly expressed low responsive TRBV family (TRBV1), less expressed TRBV families (TRBV4; 8 and 10) and responsive TRBV families (TRBV4 and 8). Many spectratypes were bell-shaped for both CD8^+^ and CD8^−^ fractions as observed for the whole T-cell population. However, differences in spectratypes appeared at this level of analysis between CD8^+^ and CD8^−^ subsets for a number of TRBV-TRBJ combinations as shown in **Figure 2A**. Indeed, CDR3 length distributions are often less regular and less complex (*i.e.* with less peaks and being more variable from fish to fish) in S2 compared to S1. Importantly, spectratyping was repeated from independent PCR amplifications and produced similar results, excluding that the expanded peaks observed in irregular profiles from S2 could be due to a random amplification of a few sequences, as frequently observed in experiments performed from cDNAs containing rare templates. To obtain a statistical validation of the differences observed between S1 and S2, we performed a quantitative analysis of all profiles using the ISEApeaks software [35]. ISEApeaks calculates the perturbation score of a given TRBV-TRBJ spectratype in comparison with a reference, summing the differences of areas calculated for each peak between the given and the reference profile [35]. Taking the average of the whole T cell population (i.e. both S1 and S2 groups), we computed the global perturbation score from all spectratypes for both S1 and S2 fractions of each individual. Principal component analysis of these perturbation values distinguishes two groups corresponding to S1 and S2, and indicates that S1 repertoires are very close to each other while S2 repertoires are much more dispersed, i.e. variable from fish to fish (**Figure 2B**). According to ebayes test, most of TRBV4-J and TRBV8-J are significantly different between both groups. A comparison of the matrices of perturbation values confirmed that TRB CDR3 length spectratypes from CD8^+^ and CD8^−^ fractions were indeed significantly different for many TRBV-TRBJ combinations especially for families TRBV4 and 8 (see **Table II**). In addition, the PCA projection of S1 and S2 samples according to the first two components (respectively capturing 58.67% and 14.94% of the global variability) confirmed that both groups are apart and showed that the inter-individual variability is higher in the S2 subset compared to S1.

**Table 2:** Statistical comparison of perturbation scores for all the TRBV-TRBJ profiles (Ebayes p-values).

	ctrl CD8^+^ vs ctrl CD8^−^	ctrl CD8^+^ vs inf CD8^+^	ctrl CD8^−^ vs inf CD8^−^
VB01C1	0,123	0,259	0,361
VB01J1-2	0,079	0,357	0,103
VB01J2	0,079	0,249	0,074
VB01J3	*0,014	0,235	0,898
VB01J4	0,074	-	0,483
VB01J6	0,146	0,622	0,730
VB01J7	*0,026	0,391	0,797
VB01J8	**0,008	-	**0,009
VB01J10	0,111	0,596	0,415
VB04C01	0,190	0,206	0,254
VB04J1-2	0,685	0,482	0,125
VB04J2	0,307	0,324	0,363
VB04J3	*0,027	**0,008	0,848
VB04J4	0,073	-	0,838
VB04J6	*0,017	0,411	0,600
VB04J7	0,149	0,116	0,814
VB04J8	*0,040	0,235	0,955
VB04J10	*0,034	0,125	0,135
VB08C01	0,538	0,088	0,742
VB08J1-2	**0,009	0,847	*0,014
VB08J2	-	0,175	**0,000
VB08J3	**0,001	**0,001	0,093
VB08J4	-	*0,011	**0,000
VB08J6	0,476	0,077	**0,000
VB08J7	*0,014	0,429	0,221
VB08J8	**0,005	0,062	0,478
VB08J10	**0,006	**0,002	**0,001
VB10C01	0,289	0,118	0,173
VB10J1-2	0,312	0,115	*0,012
VB10J2	0,174	0,292	0,095
VB10J3	0,386	0,096	**0,006
VB10J4	*0,038	*0,040	0,106
VB10J6	0,115	0,419	0,524
VB10J7	0,470	0,203	0,160
VB10J8	0,669	0,406	**0,004
VB10J10	*0,043	0,395	**0,004
#<0.05 *	14,000	5,000	10,000
#<0.01**	5,000	3,000	8,000

The degree of skewing of TRB CDR3 length distributions – the “regularity”- was objectively measured for all profiles using a diversity index derived from the Shannon index (SDI). Our adjusted Shannon diversity index (ASDI) is maximal for a high number of peaks distributed on a perfect theoretical Gaussian distribution. Comparing S1 (CD8^+^) and S2 (CD8^−^) T cell subsets, we found that the ASDI was lower for S2 compared to S1, indicating higher skewing and lower average peak numbers per profile in CD8^−^ T cells (**Figure S2A**).

Taken together, these observations indicate that the CD8^+^ T cell subset expresses a TRB repertoire more diverse and polyclonal than the CD8^−^ T cell subset(s) for which many TRB junctions show skewed spectratypes.

### CD8^+^ and CD8^–^ Lymphocyte Repertoires in VHSV-challenged Fish

To understand the respective contributions of CD8^+^ and CD8^−^ T cells to the response against a pathogen, we analysed the TRB repertoire of S1 and S2 populations from fish infected by the Viral Haemorrhagic Septicaemia Virus (VHSV), a virus causing a systemic acute infection in rainbow trout. We have shown previously that rainbow trout mounts both public and private T cell responses during secondary infections with this virus [25], [30]. However, whether the responding clones were mainly composed of CD8^+^ or CD8^−^ T cells remains unknown. To get insight into this question, clonal fish were vaccinated with an attenuated VHSV variant (strain 25–111), then subjected to a second booster injection three weeks later. Leukocytes were then harvested during the recall response and S1–3 populations were sorted as described above. The sorted fractions represented similar percentages as in control animals, and no significant differences in CD8 or CD4-1 expressions could be observed between control and infected groups (data not shown).

#### CD8^+^ TRB repertoire shows a typical response after viral infection

**Figure 3:**
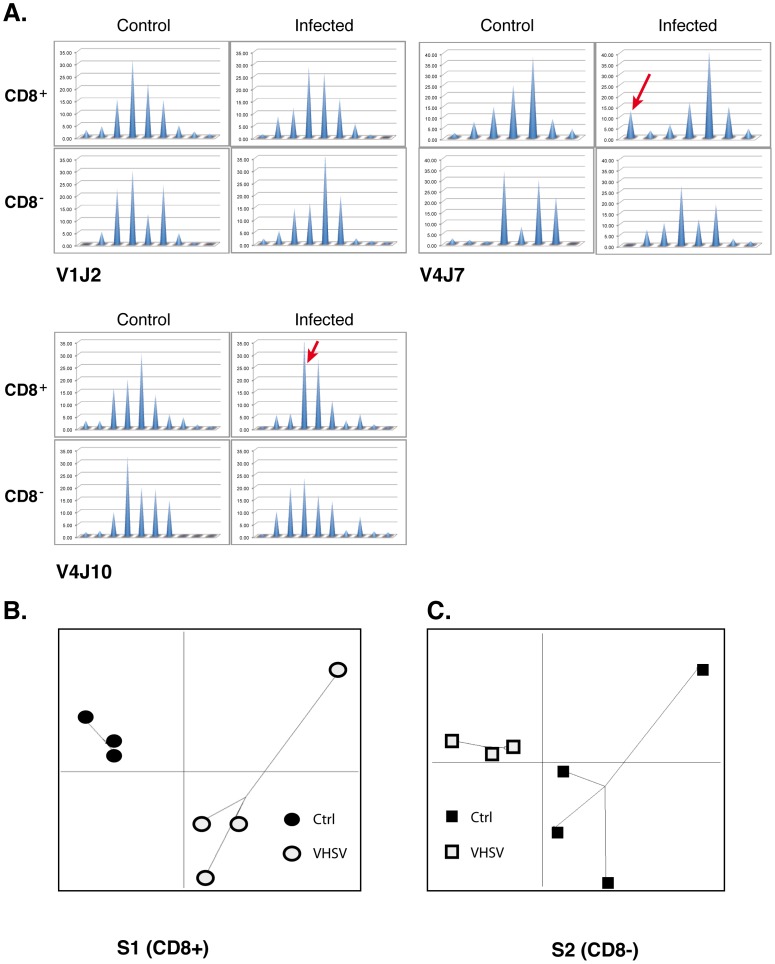
Virus infection induces modifications of the TRB repertoire expressed by CD8^+^ and CD8^−^ T cells. This figure depicts the CDR3 length distribution in CD8^+^ fractions (S1) isolated from infected and from control animals for representative TRBV-TRBJ combinations that are poorly responsive in the CD8^+^ fraction (e.g. V1J2) as well as for combinations showing CDR3 length modification upon virus infection (e.g. V4J7, V4J10). The arrows point to those peaks that represent CDR3 lengths with an increased abundance after viral infection. PCA projection of control and infected samples according to the first two components using perturbation scores calculated for all spectratypes analysed in S1 (B; PC1∶40.26% & PC2∶20.44% of the global variability) and S2 (C; PC1∶31.66% & PC2∶22.18% of the global variability) fractions.

**Figure 3A** depicts differences in CDR3 length distribution observed between CD8^+^ fractions (S1) from infected and from control animals for representative TRBV-TRBJ, *i.e.* for a combination that is poorly responsive in the CD8^+^ fraction (V1J2) as well as for combinations showing modification upon virus infection (V4J7, V4J10).

To compare the CDR3 length distributions in CD8^+^ T cells (S1 fraction) from naive and infected fish and to produce a quantitative description of spectratype modifications, we computed the perturbation score for the TRB repertoire (TRBV-TRBJ combinations) expressed by CD8^+^ T cells. For this purpose, we took the average repertoire of the CD8^+^ fraction from control fish as a reference. As illustrated by the PCA, infected and control animals are clearly distinct (**Figure 3B**). Indeed, statistical test of the perturbation matrix indicated that a number of TRBV/TRBJ profiles were significantly skewed in infected animals compared to control fish (**Table II**, see *p*-values for TRBV4/J3, TRBV8/J3, TRBV8/J4, TRBV8/J10 and TRBV10/J4). These results indicate that the TRB repertoire expressed by CD8^+^ T cells is modified by the secondary viral infection.

#### Perturbation score indicates that CD8^−^ TRB repertoire is also skewed by viral infection

We next computed the perturbation score of the CD8^−^ (S2) fractions from infected fish taking the average repertoire of the control fish as a reference. Although profile inter-individual variability in naïve animals was considerable, PCA analysis performed on these scores clearly discriminates TRB repertoires of CD8^−^ T cells from control and infected fish (**Figure 3C**). We tested differences between perturbation values of each TRBV/TRBJ profile from CD8^−^ (S2) subset in control and infected fish, respectively. We found significant differences between naïve and infected fish TRBV1/J8, TRBV8/J1-2, TRBV8/J2, TRBV8/J4, TRBV8/J6, TRBV8/J10, TRBV10/J1-2, TRBV10/J3, TRBV10/J8 and TRBV10/J10 (**Table II**). Interestingly, these combinations did not match those for which significant differences had been found for the S1 fraction.

#### Different impact of the viral infection on the TRB CDR3 length diversity in CD8^+^ and CD8^−^ T cell subsets

Selected CDR3 length spectratypes illustrate that the VHSV infection has a different impact on the TRB repertoire expressed by CD8^+^ and by CD8^−^ T cells, respectively (**Figure 3A**). To better qualify these modifications, we computed ASDIs for each profile in CD8^+^ as well as CD8^−^ fractions, using the corresponding average control repertoire as reference. While the perturbation analysis had identified unambiguous responses in CD8^+^ T cells from virus infected fish, compared to the controls, ASDIs were not significantly different between CD8^+^ T cells from infected and control fish, for any of the TRBV-TRBJ combinations considered. (**Figure S2B**).

In contrast, CD8^−^ T cells showed a different behaviour upon viral infection: the median diversity of TRB CDR3 length estimated by ASDI calculated in reference to the average repertoire of the CD8^−^ control was in fact higher in infected fish compared to the controls (**Figure S2C**). This ASDI was significantly higher (*p*-values <0.05) in infected fish for some TRBVTRBJ combinations (TRBV1/J6, TRBV4/C1, TRBV4/J1-2, TRBV4/J03, TRBV4/J10, TRBV8/J1-2; data not shown). In fact, antigen-driven clonal responses typically induce expansion of one or a few peak(s) of initially bell-shaped spectratypes, hence reduce their ASDI. In the case of the CD8^−^ fraction of trout T cells, TRB CDR3 length profiles are highly skewed in unchallenged animals and the expansion of additional virus-specific peaks therefore increases the peak diversity, thus reducing the spectratypes skew.

## Discussion

In this work, we used TRB CDR3 length profiling from CD8^+^ and CD8^−^ sorted fractions of spleen and pronephros leukocytes in order to examine the diversity and complexity of the corresponding repertoires in a teleost fish, the rainbow trout. Using anti-CD8α mabs to sort and characterize CD8^+^ and CD8^−^ T cells in different fish species, several reports have recently established that the CD8^+^ fraction comprises cytotoxic T cells, which do not express the receptor CD4, as typically known in human and in the mouse. In contrast, the CD8^−^ fraction contains CD4-1^+^ and CD4-2^+^ cells, which do not possess efficient killing capacity but have properties reminding mammalian T helper cells [14] [38]–[40]. However, the TR repertoires expressed by the CD8^+^ and CD8^−^ T cell lineages in fish remained uncharacterized so far. We therefore undertook cell sorting followed by TRB CDR3 length profiling in healthy fish as well as in animals infected by a fish rhabdovirus, the VHSV.

In PBMCs from healthy humans, TRB CDR3 length follows a bell-shaped Gaussian-like distribution for most of expressed TRBV-TRBJ combinations [27], [41]. This was also observed for both CD4^+^ and CD8^+^ subsets, although a few genes especially TRBV16 were often associated with skewed profiles [26]. Overall, the frequency of skewed profiles appeared slightly higher for CD8^+^ T cells compared to CD4^+^ T cells. This is in contrast with our observations in rainbow trout, where the CD8^+^ T cell subset expresses a TRB repertoire more diverse and more regular than the CD8^−^ fraction. We did not analyse rainbow trout PBMC because T cells are very scarce in the blood of this species, which hindered sorting experiments [42]. In fact, skewed TRB spectratypes are frequent in PBMCs of healthy macaques, not only in CD8^+^ T cells but also in CD4^+^ T cells while it is rarely seen in humans. Thus, CD4^+^ T cells were significantly contributing to the repertoire skewing in macaques, and were found for a fair number of TRBVTRBJ combinations [27]. Hence, the respective structure of the TRB diversity in CD8^+^ versus CD4^+^ T cells appears to be variable between species even among closely related ones such as primates. This observation suggests that different situations will be likely found among teleost fish, which represent a highly complex and diversified group of lower vertebrates.

Since skewed profiles were found for different TRBV-TRBJ combinations in the CD8^+^ and CD8^−^ fractions of rainbow trout lymphocytes, they should not be primarily determined by constraints at the rearrangement level. Such constraints would depend on the genes involved and would likely lead to skewed profiles in both CD8^+^ and CD8^−^ T cells. Hence, skewed profiles likely represent tracks of previous antigen-driven expansions, which are expected to affect different TRBV-TRBJ combinations in both the CD8^+^ and CD8^−^ fractions. A frequent artefact in spectratyping analysis is observed when profiles are produced from cDNAs containing very low amounts of TRB templates. In such conditions, a few sequences are randomly amplified and produce a CDR3 length profile with only one or a few peaks, which does not provide a good representation of the junction diversity [43]. To exclude the possibility that skewed spectratypes – especially from CD8^−^ cells - were due to such random amplification of rare TRB sequences, we checked the spectratypes produced from independent amplifications from the same cDNA templates. Those always showed similar patterns (data not shown) suggesting that spectratype changes reflected true TR repertoire changes. Therefore, it can be proposed that while trout CD8^+^ T cells express a highly diverse, naïve repertoire of TRB that is available to mount cytotoxic responses, the CD8^−^ T cell fraction of leukocytes would rather contain many large clones that have already accumulated during previous antigen-driven responses. We cannot completely rule out that the injection of endotoxin-free PBS to which control fish were subjected might have an impact on the TCR repertoire. However, such injection does not modify the expression level of the activation marker CTLA4 in leukocytes [44 and unpublished data], and we do not consider this is the most likely explanation to the difference between CD8+ and CD8− T cell repertoire.

Our observations are reminiscent of the accumulation of large T cell clones during aging, observed in elderly humans for both CD8^+^ and CD4^+^ lineages, leading to skewed profiles with comparable frequencies in the two subsets [29]. Alternatively, the CD8^−^ subset may be enriched in non conventional T cells with restricted TRB diversity, such as mucosal-associated invariant T (MAIT) or natural killer T (NKT)-like cells [45], [46]. Further characterization of the trout TRα repertoire will be necessary to clarify this issue.

As previously reported for the whole trout leukocyte population, we retrieved here that viral infection leads to a significant skewing of TRB CDR3 length profiles of CD8^+^ subpopulation. Both visual comparison and perturbation analysis identified TRBV-TRBJ spectratypes that are skewed after viral infection in several fish. The combinations for which skewing was significant involved TRBV4, TRBV8 and TRBV10, but not TRBV1. Interestingly, in whole-leukocyte analysis, TRBV4 has been retrieved in public responses in several trout genetic backgrounds, and TRBV8 and TRBV10 were found responsive while TRBV1 was never clearly involved in anti-VHSV response ([25], [30] and unpublished data). The diversity index did not reveal any particular pattern in infected animals; in fact, the intensity of the spectratype skewing associated to the response against VHSV in the clonal fish used in this study appeared more moderate than the modifications of the TRB repertoire we observed previously with other genetic backgrounds [25], [30], [47]. Whether this moderate response was due to the genetic background or to environmental factors is difficult to determine unambiguously, but in absence of any sign of unwanted disease in the fish used for the project we favour the first interpretation.

The analysis of the CD8^−^ response induced by the infection was more difficult due to the high frequency of skewed profiles and inter-individual variability among naïve fish regarding this fraction. However, a clear response was evidenced by the PCA, indicating that both CD8^+^ and CD8^−^ αβ T cells contribute to the anti-VHSV response. The TRBV-TRBJ combinations for which the profiles were significantly affected did not match those identified for the CD8^+^ fraction, confirming that the response to the virus does not involve cells expressing similar TRB from CD8^+^ and CD8^−^ fractions. Intriguingly, TRB CDR3 length profiles were more regular and more complex after viral infection compared to the controls, as indicated by the diversity (Shannon modified) index. It appeared that the antiviral response lead to multiple expansions of CD8^−^ T cells, sometimes reducing the relative importance of large peaks observed in the skewed profiles of the unchallenged controls. This may provide a first observation in favour of different impact of infections on CD8^−^ T cells between trout and mouse (or human). However, further investigations would be required to link these preliminary observations about CD8^+^ and CD8^−^ leukocyte fractions to the immune memory and to functional properties of cytotoxic and helper T cells in fish. In particular, the clonal complexity of CD8^+^ and CD8^−^ T cell responses will have to be characterized by a thorough sequencing analysis of their respective TRs. Also, the availability of an antibody against additional markers including CD4 would be very important to sort T cell fractions from the thymus and other lymphoid tissues and track the distribution of T cell subsets during development and after immune responses. Thus, these studies will have both basic interest for deciphering the evolution of the immune system in an important branch of vertebrates, and a practical value for improving vaccination in aquaculture.

## Supporting Information

Figure S1 **Selected TRBV/C spectratypes from S1 and S2 T cell populations purified from control rainbow trout.**
(PDF)

Figure S2 **Comparison of spectratype diversity index.** A. ASD index distribution for S1 and S2 fractions from control group. The reference used is the average repertoire through both groups. B: ASD index distribution for S1 fractions from control and infected groups. The reference used is the average repertoire of control groups. C: ASD index distribution for S2 fractions from control and infected groups. The reference used is the average repertoire of control groups.
(PDF)
