# Supplementary figures and images for: Contrasted TCRβ Diversity of CD8+ and CD8− T Cells in Rainbow Trout
Source: PLoS One. 2013 Apr 2;8(4):e60175. doi: 10.1371/journal.pone.0060175 (PMC3615082; doi:10.1371/journal.pone.0060175)

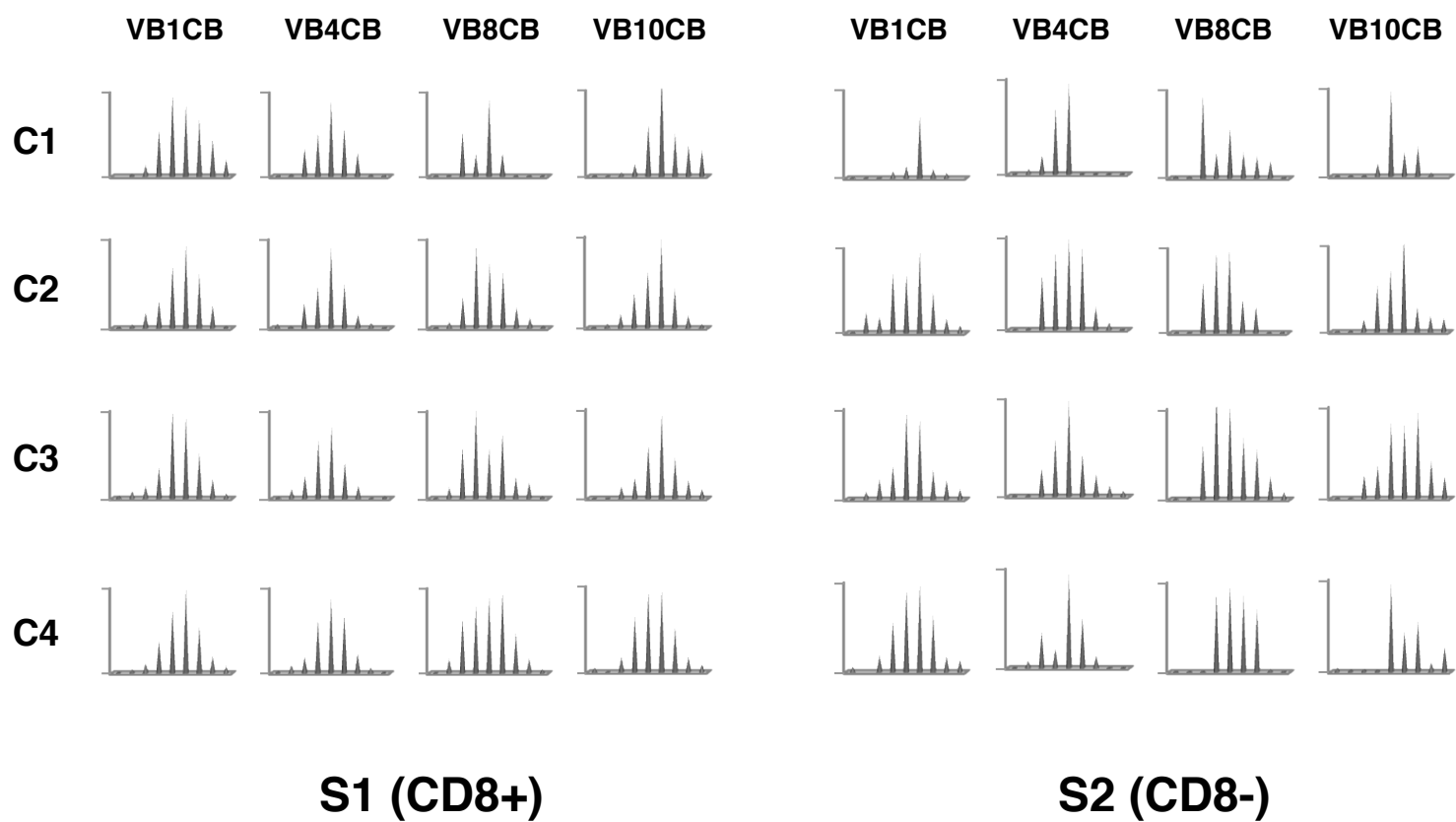

**Suppl. figure 1**

Supplement: Figure S1 — Selected TRBV/C spectratypes from S1 and S2 T cell populations purified from control rainbow trout. (PDF) [file pone.0060175.s001.pdf]
