# Supplementary material for: Contrasted TCRβ Diversity of CD8+ and CD8− T Cells in Rainbow Trout
Source: PLoS One. 2013 Apr 2;8(4):e60175. doi: 10.1371/journal.pone.0060175 (PMC3615082; doi:10.1371/journal.pone.0060175)

Supplemental Figure 2

A : ASD index distribution for S1 and S2 fractions from control group. The reference used is the average repertoire through both groups.

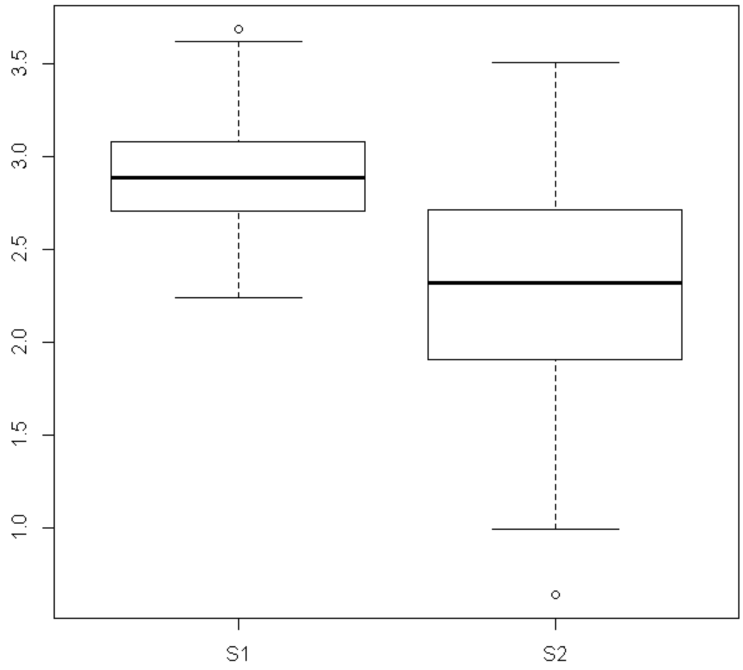

B: ASD index distribution for S1 fractions from control and infected groups. The reference used is the average repertoire of control groups.

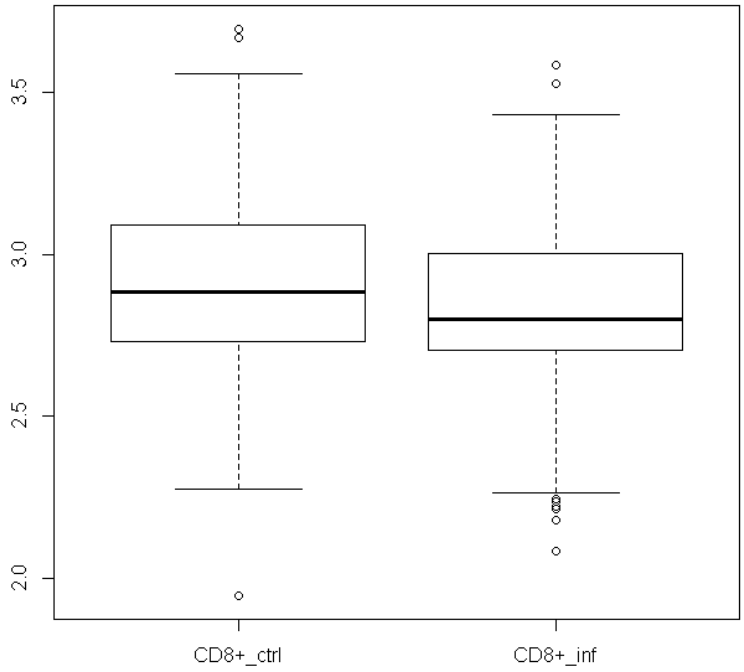

C: ASD index distribution for S2 fractions from control and infected groups. The reference used is the average repertoire of control groups.

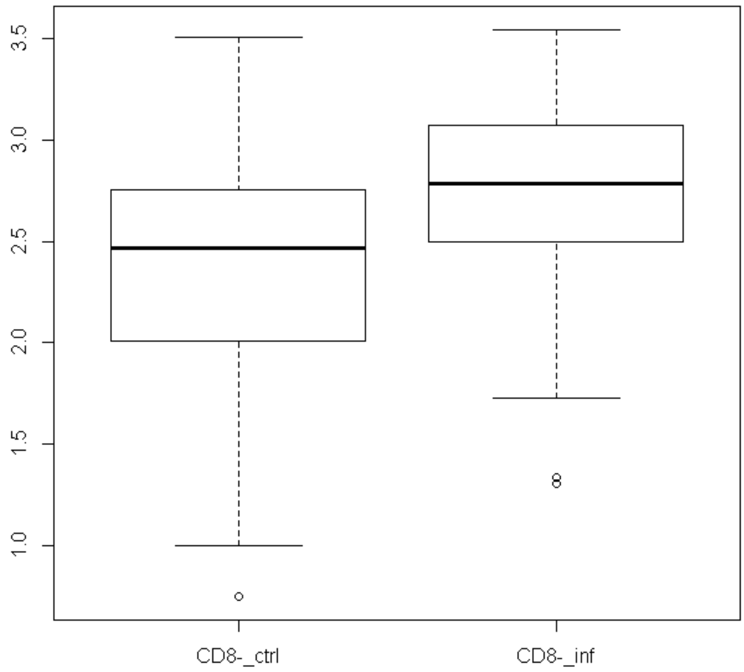

Supplement: Figure S2 — Comparison of spectratype diversity index. A. ASD index distribution for S1 and S2 fractions from control group. The reference used is the average repertoire through both groups. B: ASD index distribution for S1 fractions from control and infected groups. The reference used is the average repertoire of control groups. C: ASD index distribution for S2 fractions from control and infected groups. The reference used is the average repertoire of control groups. (PDF) [file pone.0060175.s002.pdf]
